# Incidence Trends and Geographical Variability of Pediatric Inflammatory Bowel Disease in Slovenia: A Nationwide Study

**DOI:** 10.1155/2015/921730

**Published:** 2015-11-24

**Authors:** Darja Urlep, Rok Blagus, Rok Orel

**Affiliations:** ^1^Department of Gastroenterology, Hepatology and Nutrition, University Children's Hospital, Ljubljana, Slovenia; ^2^Institute for Biostatistics and Medical Informatics, Faculty of Medicine, University of Ljubljana, Ljubljana, Slovenia

## Abstract

*Background*. The aims of the study were to determine the incidence rate of pediatric inflammatory bowel disease (PIBD) and its trends for the period of 2002–2010 and to assess the geographical distribution of PIBD in Slovenia.* Materials and Methods*. Medical records of patients (0–18 years) with newly diagnosed IBD during the study period were retrospectively reviewed.* Results*. The mean incidence rate for IBD in 2002–2010 was 7.6 per 100,000 children and adolescents per year, 4.5 for Crohn's disease (CD), 2.9 for ulcerative colitis (UC), and 0.2 for IBD-unclassified, respectively. The incidence rate increased from 5.8 per 100,000 per year in 2002–2004 to 8.6 in 2005–2007 and remained stable afterwards. Statistically significant difference in the incidence rate between the Northeastern and Southwestern parts of the country was observed (*p* = 0.025).* Conclusion*. This nationwide study demonstrates that Slovenia is among the European countries with the highest PIBD incidence. During the study period a substantial rise of PIBD incidence was observed during the first half of the study and it seems to have stabilized in the second half. The significant difference in PIBD incidence between Northeastern and Southwestern parts of the country merits further exploration of the possible environmental factors.

## 1. Introduction

Over the last decades a number of epidemiologic studies on inflammatory bowel disease (IBD) in children reported an increasing trend of pediatric incidence in developed [1–24] as well as in developing countries [25–31]. The highest incidence reported is still from the Northern countries of Europe and America. In Europe, the highest incidence of childhood IBD has been observed in Scandinavian countries and it seems that the incidence is still on the rise, mainly that of CD [18–21]. The increasing incidence of pediatric IBD (PIBD) has also been reported in the studies from other Western European countries [11–13, 22–24]. Recently, some new epidemiological data suggest that the incidence of PIBD has been increasing in Eastern Europe as well [25, 26, 28, 29].

Two epidemiologic studies on PIBD incidence were performed in Slovenia and both demonstrated an increasing trend of incidence [30, 31]. The first study was done for the period from 1994 to 2005 and covered the country's Central/Southwestern (CSW) area which is inhabited by approximately two thirds of the Slovenian pediatric population (0–18 years). It showed the mean annual PIBD incidence of 4.03 per 100,000 per year. The trend of the incidence was observed to have risen from 3.04 in the period from 1994 to 1999 to 5.14 in the period from 2000 to 2005 [30]. The second study for the period from 2002 to 2010 covered the area of the wider Northeastern Slovenia (WNE) and demonstrated a higher PIBD incidence rate of 7.6 per 100,000 children (0–18 years) per year [31].

The aims of this nationwide study were to determine the incidence rates of PIBD, Crohn's disease (CD), and ulcerative colitis (UC) and their trends for the whole country of Slovenia during the period between 2002 and 2010. Another aim was to assess the geographical distribution of PIBD throughout Slovenia.

## 2. Materials and Methods

All pediatric cases (0–18 years) of IBD newly diagnosed in the period from 2002 to 2010 and residing in well-defined statistical regions of the whole Slovenia were included in our study by a detailed retrospective review of the patients' medical records.

Although Slovenia is a small Central/Southeastern European country with only 2 million inhabitants, it is very diverse. In the North, there is the mountain range of the Alps with the appropriate mountain climate, in the East lies the Pannonian plain with the continental climate, and the Southwest side is under the influence of the Mediterranean Sea and thusly has a Mediterranean climate.

Data on the background pediatric population were obtained from the Statistical Office of the Republic of Slovenia [32]. The childhood population (0–18 years) of Slovenia was 439,448 in 2002 and 384,375 in 2010, showing a decline of 12.5% in the 9-year study period.

**Figure 1:**
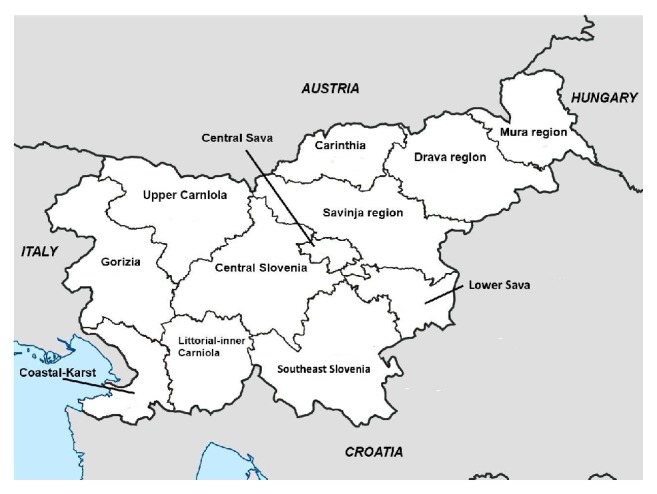
Statistical regions of Slovenia.

Due to their relatively small size, the statistical regions were grouped into three bigger units: Northeastern unit (Mura, Carinthia, and Drava regions), Central unit (Savinja, Upper Carniola, Central Slovenia, Central Sava, Lower Sava, and Southeast Slovenia regions), and Southwestern unit (Gorizia, Coastal-Karst, and Littoral-Inner Carniola regions) (Figure 1). The mean annual pediatric populations in the period of 2002–2010 (0–18 years) for Northeastern, Central, and Southwestern geographical units were 100,308, 255,623, and 52,349, respectively.

The health care system in Slovenia recommends that all children (0–18 years) with suspected IBD are to be referred to one of the two pediatric gastroenterology tertiary centres (in Ljubljana and Maribor). General pediatricians working at regional hospitals and primary health centers do not diagnose or treat patients with IBD by themselves. In Slovenia, a consensus between pediatric and adult gastroenterologists was made (both being members of the Slovenian Association for Gastroenterology and Hepatology), which states that children younger than 18 are always to be managed by pediatric and not by adult gastroenterologists. Despite the agreement, adult gastroenterologists in Slovenia were personally contacted to find out whether any young patients with IBD were under their care during the study period. There were indeed 9 adolescents under 16 years, diagnosed by adult gastroenterologists; however, they were then referred to a pediatric centre for appropriate treatment and were included in the study.

Newly diagnosed cases of IBD (0–18 years) were retrospectively identified based on the admission register and the endoscopic register. In the admission register run at the gastroenterology unit (both for inpatients and outpatients) only the data on diagnoses (IBD without subtypes), names, surnames, dates of birth, dates of diagnosis, and the individual inpatients' and outpatients' numbers were recorded. Based on these data we were able to find the medical inpatient and/or outpatient records which provided us with other required information. Because we were not sure that all newly diagnosed patients with IBD were indeed recorded at admission we also reviewed the endoscopic register, where all upper and lower endoscopies performed, along with their endoscopic results, are recorded. Such an endoscopic register must necessarily be maintained due to the Slovenian health insurance policy. We obtained all required data (pathohistological, clinical data and data on the small bowel investigations and additional endoscopic investigations) from the patients' medical records. Only those patients with a confirmed diagnosis of IBD were included in our study.

The diagnosis of IBD was based on clinical signs and symptoms, a physical examination, endoscopic appearance, histological assessment of mucosal biopsy specimens, and small bowel imaging studies. Patients who did not fulfill the diagnostic criteria for IBD regarding the macroscopic changes at endoscopy and typical histological findings were excluded from the study. The diagnosis was never based only on clinical presentation and radiological or/and ultrasound findings. Data from the patients' medical records about diagnostic workup were compared with the Porto diagnostic criteria (Table 1) [33].

Disease location according to the Paris classification was evaluated only in patients with CD and UC with a complete diagnostic workup [34]. The complete diagnostic workup consisted of upper GI endoscopy and the small bowel investigations (small bowel follow-through, capsule endoscopy, MR enterography, or CT imaging of small bowel). Capsule endoscopy was performed in all of the patients with suspected or diagnosed CD after the year 2006 as part of the routine diagnostic workup. Only reliable changes (aphthous lesions, ulcers, and pseudopolyps) were assessed in order to define the localization of inflammation and therefore the extent of the disease. CD patients with pathological findings of the small bowel observed only by abdominal ultrasound imaging were not included in the evaluation of disease location.

The disease extent in the colon was defined only by macroscopic lesions visible during endoscopy, whereas the involvement of the upper GI tract was defined by the presence of either typical macroscopic changes or highly specific microscopic lesions (granulomas).

Incidence rates of total IBD, CD, UC, and IBD-U were calculated for whole Slovenia, for each of the twelve statistical regions, and for the three aforementioned geographical units. Mean annual incidences for the three time periods (2002–2004, 2005–2007, and 2008–2010, resp.) were determined as well.

### 2.1. Statistical Analysis

The population data for the period from 2002 to 2010 were obtained by the Statistical Office of the Republic of Slovenia (SURS) [32]. Incidence rates were calculated as events per 100,000 person-years and their 95% confidence intervals (CI) were based on the Poisson distribution.

The differences between periods or regions were estimated with the Poisson model, where the number of events was considered as a dependent variable and the population size was included in the model as an offset term.

Starting from 31 December 2008 the Statistical Office began to use a different definition of the population which reduced the number of inhabitants by around 1%. It is estimated that this decrease occurred mainly due to not including foreigners who live in Slovenia for less than one year, which are in majority estimated to be over 18 years of age, suggesting that this change of the definition only had a marginal effect on our study population. It should be noted however that it is still possible that due to the change in the methodology the incidence rates in the period from 2009 to 2010 can be slightly overinflated in comparison with 2002–2008.

The study was approved by the National Medical Ethics Committee.

## 3. Results

**Figure 2:**
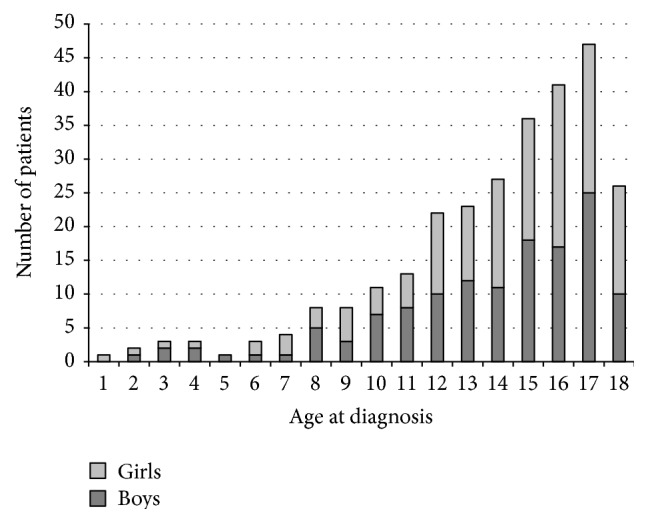
Age at diagnosis for girls and boys with IBD.

In total, 279 cases of IBD in Slovenian childhood and adolescent population (0–18 years) were newly diagnosed during the study period from 2002 to 2010 (167 CD, 105 UC, and 7 IBD-U). The median age at IBD diagnosis was 13.7 years (range: 1.5–18) (Figure 2). The female/male ratio was 1.1, 1.2, and 0.8 for CD, UC, and IBD-U, respectively.

Of 279 newly diagnosed IBD patients, 89/279 (31.9%) were treated at Maribor's centre, 188/279 (67.7%) of them were treated at Ljubljana's centre, and 2/279 (0.7%) patients were treated at both centers.

The main characteristics of IBD patients are shown in Table 2.

### 3.1. Nationwide Incidence Rates

The mean annual incidence per 100,000 children and adolescents between 0 and 18 years of age for entire Slovenia in the period from 2002 to 2010 was 7.59 (95% CI: 6.72–8.53) for all IBD, 4.54 (95% CI: 3.88–5.28) for CD, 2.85 (95% CI: 2.33–3.45) for UC, and 0.19 (95% CI: 0.07–0.39) for IBD-U, respectively.

The nationwide incidence rates of total IBD, CD, UC, and IBD-U and their 95% CI for each year of the study period are presented in Table 3.

**Figure 3:**
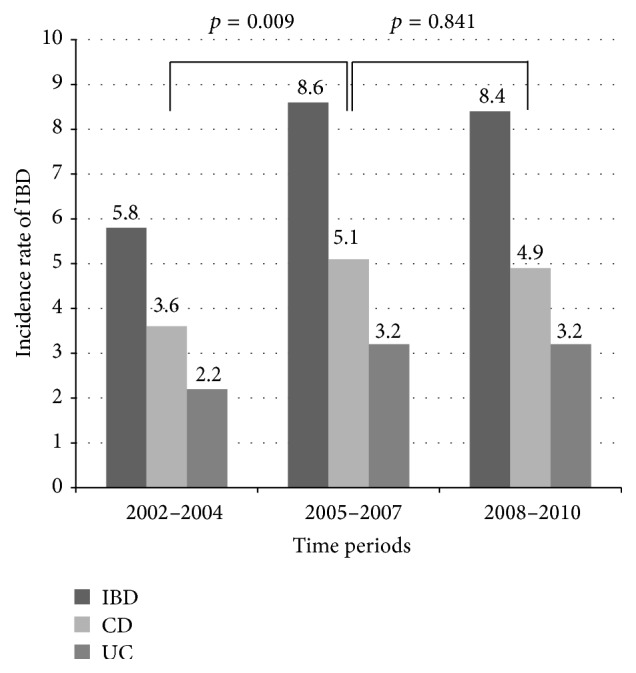
Incidence rates for pediatric IBD, CD, and UC per 100,000 children and adolescents for 3-year periods per year in 2002–2010.

PIBD incidence rates for 3-year periods are shown in Figure 3. There was a statistical significant increase in the incidence rate of PIBD between the periods from 2005 to 2007 and from 2002 to 2004 (*p* = 0.009) and the periods from 2008 to 2010 and from 2002 to 2004 (*p* = 0.017); however, no statistical significant difference was seen between the periods from 2005 to 2007 and from 2008 to 2010 (*p* = 0.841).

### 3.2. Geographical Distribution of the PIBD Incidence Rates in Slovenia

**Figure 4:**
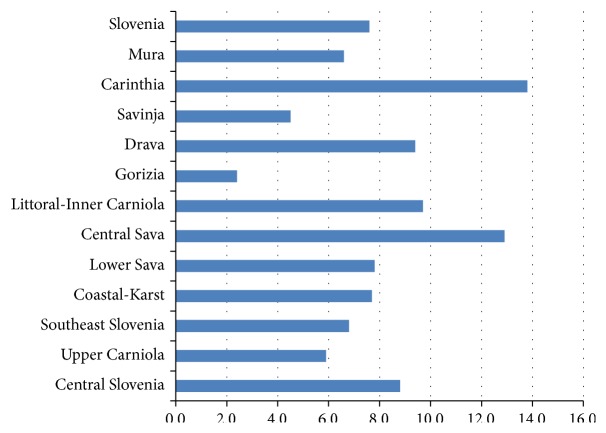
Twelve Slovenian statistical regions and mean annual PIBD incidence rates (per 100,000 children and adolescents) in 2002–2010.

The mean annual incidence rates of PIBD for the twelve Slovenian statistical regions for the whole period of 2002–2010 are presented in Figure 4. The highest PIBD incidence of 13.8 per 100,000 per year was observed in Carinthia, which is the most Northern Slovenian statistical region, positioned along the border with Austria. On the contrary, the lowest incidence rate of 2.4 was seen in the most Western Gorizia region bordering Italy.

**Figure 5:**
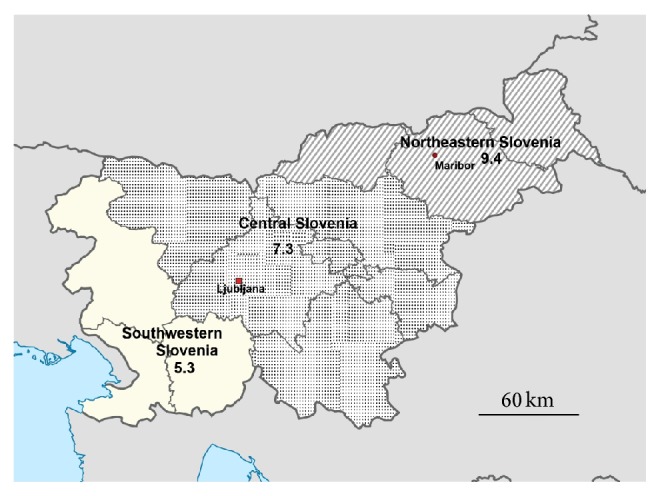
Three geographical units of Slovenia and mean annual PIBD incidence rates (per 100,000 children and adolescents) in 2002–2010.

When the three bigger regional units were taken into account, the highest incidence of PIBD was found to be in the Northeastern Slovenia (9.4 per 100,000 per year) and the lowest in the Southwestern Slovenia (5.7 per 100,000 per year), while the incidence in Central Slovenia was between the aforementioned two (7.3 per 100,000 per year) (Figure 5). There was a statistically significant difference in the mean annual incidence rate of PIBD between the Northeastern and Southwestern regional units (incidence ratio (IR) 1.64, 95% CI from 1.07 to 2.53, and *p* = 0.025) but not between the Central unit and any of these two (IR = 0.77, 95% CI from 0.59 to 1.01, and *p* = 0.051 and IR = 1.27, 95% CI from 0.84 to 1.91, and *p* = 0.255 for the comparison with the Northeastern Slovenia and Southwestern Slovenia, resp.). Similar differences between Northeastern Slovenia, Central Slovenia, and Southwestern Slovenia were found for the incidences of CD and UC, but they were not statistically significant (*p* > 0.05) (Table 4).

## 4. Discussion

This study is the third on the incidence rate of PIBD in Slovenia, but it is the first nationwide study covering the whole country.

Two previous studies were conducted in two different time periods and for different geographical regions of Slovenia. The first study was made for the period of 1994–2005 and only for the CSW Slovenia. The observed mean annual PIBD incidence in the 1994–2005 study was 4.03 per 100,000 per year [30]. The second study did cover the area of WNE but it also included the Savinja statistical region which is closer to Central Slovenia than Northeastern statistical region from the geographical view. The second study showed a higher incidence rate of 7.6 per 100,000 per year in comparison with the first one [31]. Partially different time periods and different geographical areas did not allow us to draw any conclusions on whether there has been a real rise in the PIBD incidence in Slovenia in the last years.

Surprisingly, the current study covering whole Slovenia has demonstrated the mean annual PIBD incidence rate of 7.6 per 100,000 per year, which is the same incidence that was reported in the previous study that covered only the WNE (wide Northeastern Slovenia) for the same time period. This observation has confirmed that PIBD throughout whole Slovenia is high and comparable to the reported incidences from Western European countries [12, 19, 22–24]. During the study period a substantial rise of PIBD incidence was observed during the first half of the study and it seems to have stabilized in the second half. Whether the incidence of PIBD in Slovenia has already reached a plateau is yet to be discovered. Therefore, more prospective epidemiologic data are needed along with a nationwide PIBD register.

This study has shown that the PIBD incidence varies greatly among different statistical regions. The results are biased by the relatively small number of newly diagnosed IBD patients per year and a small population number in each region as well. However, when the three bigger geographical units were taken into account, a statistically significant difference in PIBD between Northeastern (NE) Slovenia and Southwestern (SW) Slovenia was found. The incidence of PIBD observed in NE Slovenia was 9.4 per 100,000 per year and the one in SW Slovenia was 5.7 per 100,000 per year. We can only speculate that this difference is at least partially due to the regions being affected by the different climate conditions. In the NE Slovenia, which borders Austria and Hungary, a subalpine and continental climate is present in contrast to the typical Mediterranean climate with abundant sunlight exposure over the whole year in SW Slovenia.

The North-South gradient has long been known for IBD [35, 36]. In several countries including UK, France, USA, and Finland, North-South gradients have been reported as well [11, 37–39]. Recently, several studies have pointed out an important immunological role of vitamin D and its association with autoimmune diseases such as IBD [40–44].

Although there are some indicators that may explain the role of sunlight exposure in the observed difference between the most Northeastern and Southwestern parts of the country, we believe that there may be some other more important environmental factors that contribute to the observed geographical difference of the PIBD incidence in Slovenia.

Northeastern regions of Slovenia are known for their traditional continental diet which is characterized by a large amount of saturated fats and refined carbohydrates with a high content of red meat (mainly pork) and a consequently high content of omega-6 fatty acids. On the contrary, the population of the Southwestern part of the country is accustomed to the Mediterranean diet with large amount of fish, olive oil, vegetables, and fruits and therefore higher amounts of fiber and omega-3 fatty acids and lower amounts of saturated fats. Several studies have pointed out the role of a diet as a possible risk factor for IBD development [45–52]. There is no doubt that genetic factors may also account for some of the observed differences in the PIBD incidence between NE Slovenia and SW Slovenia; therefore studies on the genetical background of these two Slovenian populations could be of importance. On the other hand, NE Slovenia borders Austria and Hungary and thusly the NE Slovenian population has a similar cuisine to that of those two neighbouring countries, with a typical heavy, high fat continental diet largely resulting from the historically common Austro-Hungarian diet. On the contrary, the cuisine in the SW parts of the country is similar to the Italian Mediterranean cuisine. It has been postulated that there is an important interplay between genetic and environmental factors which are involved in the IBD aetiopathogenesis. Dietary factors may influence the expression of genes involved in the IBD pathogenesis, and the genetic background determines which environmental factors may have a role in the development of IBD in genetically predisposed individuals [53, 54].

This study has shown that PIBD incidence in Central Slovenia is lower when compared to NE Slovenia. This observation is in contrast with our expectations, because the Central parts of Slovenia are economically more developed, and the standard of living is higher when compared to the Northeastern part. Therefore, our study did not confirm the hypothesis that economic development is a potential risk factor for IBD.

Although we are aware that this study shares some of the disadvantages of a retrospective approach, we believe it is representative, mainly because of the consistency of our healthcare system with only two tertiary pediatric gastroenterology centers, where all pediatric IBD patients up to 18 years of age are to be diagnosed and treated. Nevertheless there may be some patients below 18 years of age who may have been diagnosed by adult gastroenterologists. Therefore, our data on the PIBD incidence may be slightly underestimated and must therefore be interpreted with caution considering the retrospective design of the study.

On the other hand, this study is one of the rare studies that were truly nationwide and it covered the whole population of the country.

## 5. Conclusion

This study has demonstrated a high incidence rate of PIBD in Slovenia. During the study period a substantial rise of the PIBD incidence was observed only during the first half of the study and it seems to have stabilized in the second half. Whether the incidence of PIBD in Slovenia has already reached a plateau is yet to be discovered.

The significant difference in PIBD incidence rates between the most Northeastern and Southwestern regions of Slovenia merits further exploration. Prospective epidemiological studies are needed to clarify the role of environmental factors in the pediatric inflammatory bowel disease in Slovenia and worldwide.

## Figures and Tables

**Table 1:** Diagnostic procedures performed at diagnosis.

	CD (*n* = 167)	UC (*n* = 105)	IBD-U (*n* = 7)
Colonoscopy	167 (100%)	105 (100%)	7 (100%)
Terminal ileocolonoscopy	153 (90.3%)	91 (86.4%)	6 (85.8%)
Upper GI endoscopy	158 (93.2%)	81 (76.9%)	7 (100%)
Abdominal ultrasound	167 (100%)	105 (100%)	7 (100%)
Small bowel follow-through	42 (24.7%)	5 (4.7%)	0 (0%)
Scintigraphy with labeled leucocytes	21 (12.4%)	8 (7.6%)	0 (0%)
Capsule endoscopy^*∗*^	87 (51.3%)	11 (10.4%)	4 (57.2%)
Magnetic resonance imaging (MRI)^+^	18 (10.6%)	2 (1.9%)	2 (28.6%)
Computed tomography (CT)	5 (2.9%)	0 (0%)	0 (0%)

^*∗*^Capsule endoscopy has been available since 2006.

^+^MR enterography has been available since 2009.

**Table 2:** IBD patient characteristics (*n* = 279).

	*n*	%
Female	145	52
Male	134	48
Age at diagnosis (y)		
0–4	9	3.2
5–9	24	8.6
10–14	96	34.4
15–18	150	53.7
Positive family history	44	15.7
Location of CD (Paris)		
CD patients with available data	112	66
L1 ileal/ileocecal	21	18.7
L2 colon	11	9.8
L3 ileocolonic	24	21.4
L4a gastric	2	1.8
L4b proximal ileum and jejunum	1	0.9
L1 + L4a	5	4.5
L1 + L4b	6	5.3
L3 + L4a	26	23.1
L3 + L4b	11	9.8
L3 + L4a + L4b	5	4.5
Location of UC (Paris)		
E4 pancolitis	68	64.6
E3 distal to hepatic flexure	19	18.1
E2 left-sided colitis	13	12.35
E1 proctitis	5	4.75
Behaviour of CD		
B1 inflammatory	143	85.7
B2 stricturing	10	6.0
B3 fistulizing	14	8.3

**Table 3:** Annual incidence (per 100,000, 95% CI) of IBD, CD, and UC among children and adolescents with 0–18 years of age in Slovenia, 2002–2010.

Annual incidence	IBD (*n* = 279)	CD (*n* = 167)	UC (*n* = 105)
2002	5.7 (3.7–8.4)	3.6 (2.1–5.9)	2.0 (0.9–3.9)
2003	6.1 (3.9–8.9)	3.7 (2.1–6.1)	2.3 (1.1–4.3)
2004	5.7 (3.7–8.5)	3.6 (1.9–5.9)	2.1 (0.9–4.0)
2005	6.8 (4.5–9.8)	3.9 (2.2–6.3)	2.9 (1.5–5.1)
2006	9.4 (6.6–12.9)	5.7 (3.6–8.5)	3.5 (1.9–5.8)
2007	9.7 (6.9–13.3)	5.7 (3.6–8.6)	3.2 (1.7–5.5)
2008	7.3 (4.9–10.5)	5.1 (3.1–7.8)	2.0 (0.9–3.9)
2009	8.4 (5.8–11.8)	5.1 (3.1–7.9)	3.3 (1.8–5.7)
2010	9.4 (6.6–12.9)	4.6 (2.7–7.2)	4.3 (2.5–6.9)

**Table 4:** Mean annual incidence (per 100,000, 95% CI) of IBD, CD, and UC among children and adolescents in the three Slovenian geographical units, 2002–2012.

	IBD	CD	UC
Northeastern Slovenia	9.4 (7.5–11.6)	5.6 (4.2–7.4)	3.4 (2.3–4.8)
Central Slovenia	7.3 (6.2–8.4)	4.3 (3.5–5.3)	2.7 (2.1–3.5)
Southwestern Slovenia	5.7 (3.8–8.3)	3.4 (1.9–5.5)	2.3 (1.2–4.2)
